# The Practice of Medicine in Georgia

**Published:** 1893-09

**Authors:** Arthur C. Blain

**Affiliations:** Macon, Ga.


ATLANTA

MEDICAL AND SURGICAL JOURNAL.

VOL. X. SEPTEMBER, 1893. No. 7.

EDITED BY

LUTHER B. GRANDY, M. D., AND MILLER B. HUTCHINS, M. D.,

WITH THE CO-OPERATION OF

H. V. M. MILLER, M. D., LL. D., VIRGIL O. HARDON, M. D.,

FLOYD W. McRAE, M. D., AND ARTHUR G. HOBBS, M. D.

### ORIGINAL COMMUNICATIONS.

### THE PRACTICE OF MEDICINE IN GEORGIA.

BY ARTHUR C. BLAIN, B. C. S., M. D.,
MACON, GA.

It is my purpose today to call your attention to the laws, or rather the want of laws, governing the practice of medicine in this State. Now, it is simply necessary for a man to have a diploma from a chartered medical college and register the same with the clerk of the superior court in the county where he expects to practice. In the majority of cases, I venture to say that you could have any kind of diploma written in Latin, and tell the clerk that it is your medical diploma and he would readily register you; thus giving the full sanction of the law to frauds, quacks and ignoramuses, who may have gained the sanction of some mercenary college faculty, who certify to their fitness to practice the healing art, when they are about as much fitted for it as a horse is for an undertaker. In fact, while the quacks and frauds are usually men of sufficient sense to use only such remedies and other measures as are perfectly bland, the latter class, who have only a smattering knowledge of medicine, are liable to do damage to the community that is unfortunate enough to be imposed upon by them.

> "A little learning is a dangerous thing.
> Drink deep, or taste not the Pierian spring."

Now let us place ourselves in the shoes of the public, who are not competent to judge of the learning of the physician and have to rely solely upon the licenses of the individual, and the (I am sorry to say it) too often empty title of Doctor of Medicine. Just suppose, gentlemen, that in an emergency obstetrical or surgical case you were to call in one of these, because he happened to be the first doctor you could find (or probably the only one), to attend your wife, mother or sister in a life and death case, and probably too late find out your mistake; would you not censure the laws permitting such impostors to practice among you? I say without hesitation that you would.

We, as true physicians, are derelict in our duty if we do not awake and bestir ourselves in an effort to remedy this great defect, and we can only accomplish this end by a continued and united struggle to place this matter in its proper light before the legislators, and when they once understand the enormity of the evil, I venture to say that the required legislation would not long remain wanting. Let us turn to the Code of Ethics and refer to the duties of the profession to the public. Chapter 3, article 1, section 4, says: "It is the duty of the physicians who are frequently witnesses of the enormities committed by quackery, and the injury to health and even destruction of life caused by the use of quack medicines, to enlighten the public on these subjects, to expose the injuries sustained by the unwary from the devices and pretensions of artful empirics and impostors," etc. Let us labor to follow this mandate by gaining statutory enactments which will prevent these impostors.

In this country where liberty is confounded with unbridled license; where justice is often an article of merchandise; where science is used as a cloak to cover ignorance; where, in short, quackery, humbuggery and criminality flourish like vegetation in the tropics, in such a land, what we need is proper laws for the protection of the people as well as ourselves. Laws, that while recognizing and protecting the rights of all educated physicians without regard to their creeds, or modes of treatment, would effectually uproot and weed out the whole line of quacks, abortionists, faith-curists and ignoramuses, who know as little about the practice of medicine, and attributes of a true physician as they do about the world to come.

We want medical laws that discriminate in favor of the true men of science and integrity, and against the empiric and impostor. Such laws are essential to public health and safety.

In the absence of any further law in our State, it would be far better were our medical diplomas written in plain English, so that the clerks in our courts who do not happen to be extra good Latin scholars could not be gulled by bogus diplomas.

Let our laws be founded on the motto of our State, Wisdom, Justice and Moderation; although I would not insist on a too close adherence to the latter.

Now, the remedy for the existing evil is the passage of the proper legislation regulating the practice of medicine in Georgia and creating a State Board of Medical Examiners. The dentists of our State have a similar law, and why should we, whose knowledge or ignorance may save or destroy human life, be debarred? It is a blot on the face of our State that she has no such law, when her sister States are protected; and Georgia is made a dumping ground for all who fail to pass the requisite examination to obtain a license to practice in Virginia, Florida or Alabama.

The time is ripe for such legislation, and I repeat that we must work constantly and in unison to obtain the desired end, remembering that the constant dripping of water will wear away the solid rock; and I believe that when the enormity of the situation is properly brought before our legislators, that they will not long permit this stigma to remain upon the Empire State of the South, and she will no longer be an asylum for quackery and all medical impostors.

I have before me reports for 1892 from the examining boards of

Alabama and Virginia showing that out of six hundred and forty-seven applicants eighty-eight were rejected as unfit to practice in those States. Now, of course our State was a natural dumping ground for these abortions. I also regret to see from these reports the unfavorable showing made by some of our Georgia colleges.

State boards not only afford protection to the people from charlatans and unqualified practitioners, but exert a salutary influence towards elevating the educational standard of the medical profession.

We would do well to copy to a great extent the excellent law of the State of Minnesota, which, I believe, is conceded to be the best in the union, taking care to make the board non-partisan, and inserting a clause making all professors in medical colleges ineligible, thus freeing it entirely of all bias in favor of any medical college. Mixed examining boards are also objectionable; better to have separate boards of seven each, a board of regular physicians, of Homeopaths and one of Eclectics, as is the case with the present law of Maryland. More than two thousand M. D.'s are now graduated annually in our country, while even now there is one doctor for about every six hundred souls in the union. Diplomas are now issued too freely to make them of very great value in the estimation of the public, and are not received as evidence of their owner's competency, either by the army or naval examining boards, or even by State licensing boards.

The doors of the Æsculapian temples hang too much ajar and invite all to rush in; however, I am glad to note the tendency of many of our medical colleges to raise the requirements both for entrance and graduation.

It would be a wise move on the part of this association to send a committee to Atlanta during the next session of the legislature to assist in formulating and passing some good law that would meet the requirements of the case. I believe it would be a good idea, were it possible, to pass a law making it compulsory upon every regular physician practicing within this State to become a member of the Medical Association of Georgia—constituting our Board of Censors the State Board of Examiners; and all who failed to pass the necessary examinations on anatomy, physiology, chemistry, pathology and practice, obstetrics and gynecology, materia medica and therapeutics and surgery would be debarred from the society and consequently from license to practice in the State. There is still one other matter to which I wish to direct your attention, and like the foregoing it, too, requires creation by our lawmakers. I refer to a State Board of Health.

Let us again turn to our Code of Ethics and see what we can find there touching this subject. Chapter 3, article 1, section 1, says: "As good citizens, it is the duty of physicians to be ever vigilant for the welfare of the community, and to bear their part in sustaining its institutions and burdens; they should also be ever ready to give counsel to the public in relation to matters especially appertaining to their profession, as on subjects of medical police, public hygiene and legal medicine. It is their province to enlighten the public in regard to quarantine regulations, the location, arrangement and distances of hospitals, asylums, schools, prisons and similar institutions; in relation to the medical police of towns, as drainage, ventilation, etc.; and in regard to measures for the prevention of epidemic and contagious diseases; and when pestilence prevails it is their duty to face the danger, and to continue their labors for the alleviation of the suffering, even at the jeopardy of their own lives."

Our duty to the healthy is as great as to the diseased. Indeed, the protection of the public health is of far greater importance than the well-being of any individual. I have carefully examined the bill introduced at the last session of the legislature by Judge Mershon, of Glynn county, entitled "An act to create and maintain a State Board of Health," and find that this can be made an excellent measure by a few changes. I understand that the objection urged against it at the session was that the bill gave the health officers too much authority, when, in my opinion, that is the most important feature of the measure, for if the health officer does not have the necessary authority to act quickly he will be a mere figurehead.

Let us have a committee who will urge the passage of this or some similar measure, using all the powers which they possess to engineer it through. Some of you may urge that a few years since our association had a similar committee, which failed in its objects, and therefore it would be useless to appoint another, but I say let us follow the schoolboy's lines: "If at first you don't succeed, try, try again." Besides, I think now we have a far more intelligent legislature than we had then. The time seems to be a propitious one now, from the danger of an outbreak of cholera in this country at any moment.

May we be able to wield the desired influence over our lawmakers, and if these remarks tend in any way to assist in awakening our populace to the urgent necessity of such laws, I will consider my time well expended.

